# Inorganic phosphate and the risk of cancer in the Swedish AMORIS study

**DOI:** 10.1186/1471-2407-13-257

**Published:** 2013-05-24

**Authors:** Wahyu Wulaningsih, Karl Michaelsson, Hans Garmo, Niklas Hammar, Ingmar Jungner, Göran Walldius, Lars Holmberg, Mieke Van Hemelrijck

**Affiliations:** 1 King’s College London, School of Medicine, Division of Cancer Studies, Cancer Epidemiology Unit, London, UK; 2 Department of Surgical Sciences, Uppsala University, Uppsala, Sweden; 3 Regional Cancer Centre, Uppsala University Hospital, Uppsala, Sweden; 4 Department of Epidemiology, Insitute of Environmental Medicine, Karolinska Institutet, Stockholm, Sweden; 5 AstraZeneca Sverige, Södertalje, Sweden; 6 Department of Medicine, Clinical Epidemiological Unit, Karolinska Institutet and CALAB Research, Stockholm, Sweden

**Keywords:** Cancer, Inorganic phosphate, Prospective cohort study

## Abstract

**Background:**

Both dietary and serum levels of inorganic phosphate (Pi) have been linked to development of cancer in experimental studies. This is the first population-based study investigating the relation between serum Pi and risk of cancer in humans.

**Methods:**

From the Swedish Apolipoprotein Mortality Risk (AMORIS) study, we selected all participants (> 20 years old) with baseline measurements of serum Pi, calcium, alkaline phosphatase, glucose, and creatinine (n = 397,292). Multivariable Cox proportional hazards regression analyses were used to assess serum Pi in relation to overall cancer risk. Similar analyses were performed for specific cancer sites.

**Results:**

We found a higher overall cancer risk with increasing Pi levels in men ( HR: 1.02 (95% CI: 1.00-1.04) for every SD increase in Pi), and a negative association in women (HR: 0.97 (95% CI: 0.96-0.99) for every SD increase in Pi). Further analyses for specific cancer sites showed a positive link between Pi quartiles and the risk of cancer of the pancreas, lung, thyroid gland and bone in men, and cancer of the oesophagus, lung, and nonmelanoma skin cancer in women. Conversely, the risks for developing breast and endometrial cancer as well as other endocrine cancer in both men and women were lower in those with higher Pi levels.

**Conclusions:**

Abnormal Pi levels are related to development of cancer. Furthermore, the in verse association between Pi levels and risk of breast, endometrial and other endocrine cancers may indicate the role of hormonal factors in the relation between Pi metabolism and cancer.

## Background

Dietary patterns are suggested to be an important environmental risk factor for cancer [1]. Inorganic phosphate (Pi) is a dietary constituent well-known for its role in skeletal mineralization, and normal levels of Pi are essential to maintain normal cellular function [2]. Recent experimental studies in rodents indicated that Pi may act as an active regulator of growth rather than a merely compulsory element in cellular homeostasis. Elevated levels of serum Pi were found to modify gene expression as well as protein translation and affect the rate of cell proliferation *in vitro*[3,4]. Moreover, a high Pi diet has been reported to result in a significantly increased development of lung and skin cancers, as well as perturbed normal brain growth in animal studies [5-7], which denoted the potential link between Pi and carcinogenesis in humans. However, to our knowledge there are no observational studies describing the association between Pi and cancer risk in humans.

Besides being naturally present in raw food including meats, fish, eggs, dairy products and vegetables, Pi is also found as an additive in processed food such as hamburgers and pizza, and as phosphoric acid in soda beverages [8]. Mostly, this Pi content is not listed as an ingredient per se, and it was reported that this ‘hidden’ Pi content of food with Pi-containing additives is nearly 70% higher than in food without additives [9]. In the human body Pi is known to be mainly regulated by a set of hormonal and metabolic factors which tightly control calcium homeostasis, i.e. vitamin D and parathyroid hormone (PTH), and a recently identified Pi-regulating hormone, fibroblast growth factor 23 (FGF-23). However, intestinal absorption of Pi is efficient and minimally regulated [2,10], so that high Pi supplementation results in markedly elevated levels of serum Pi [11,12]. Additionally, abnormal Pi levels are also a common feature of various metabolic diseases including diabetes and rickets [13,14]. Considering the emerging experimental evidence linking Pi and cancer, it is of interest to explore this relation in an observational population-based setting.

## Methods

### Study population and data collection

The Swedish Apolipoprotein Mortality Risk (AMORIS) database has been described in detail elsewhere [15-17]. Briefly, this database is based on the linkage of the Central Automation Laboratory (CALAB) database (1985–1996) to various Swedish national registries, including the National Cancer Register. The CALAB database includes data from 351,487 male and 338,101 female individuals having clinical laboratory testing as part of a general health check-up or outpatients referred for laboratory testing. No individuals were inpatients at the time their blood samples were taken. This study complied with the Declaration of Helsinki, and the ethics review board of the Karolinska Institutet approved the study (diary number: 2010/1047-31/1).

We selected all participants aged 20 or older with baseline measurements of serum Pi, calcium (mmol/L), alkaline phosphatase (mmol/L), glucose (mmol/L) and creatinine (μmol/L) (n = 397,292). All participants were free from cancer at time of entry and none were diagnosed with cancer or died within three months after study entry. Follow-up time was defined as the time from measurement until date of cancer diagnosis, emigration, death, or study closing date (31^st^ of December 2002), whichever occurred first. The CALAB database also contained information on age, season at time of measurement, and fasting status. Diagnosis of cancer were obtained from the National Cancer Register and classified based on the International Classification of Diseases, seventh revision (ICD-7; codes for specific cancer sites are presented in tables). Socioeconomic status (SES) was taken from the consecutive Swedish censuses during 1970–1990 and is based on occupational groups and classifies gainfully employed subjects into manual workers and non-manual employees, below designated as blue-collar and white-collar workers [18]. History of hospitalization for diabetes (ICD-7: 260) and lung disease (ICD-7: 470–527; mostly include upper and lower respiratory tract infections and did not include asthma) was obtained from the National Patient Register.

Serum inorganic phosphate was measured via formation of the phosphomolybdic acid complex (coefficient of variation ≤4%) [19]. To assess the effect of small changes in serum Pi levels, we calculated standardized values of Pi using its standard deviation (SD) as a unit. Calcium and alkaline phosphatase were measured by colorimetric method [20,21], while glucose was measured enzymatically with a glucose-oxidase/peroxidase method [22]. Serum creatinine was measured with the Jaffé method (kinetic) [23]. All laboratory examinations were performed using described methods above with automated and calibrated instruments in the same laboratory [24].

### Data analysis

Multivariate Cox proportional hazards models were used to investigate quartiles and standardized values of serum Pi as a continuous variable in relation to overall incident cancer. All models were adjusted for age, gender and SES. We also took into account serum glucose, fasting status and history of diabetes based on hospital discharge diagnosis since diabetes is known to modify the risk of cancer and Pi metabolism is abnormal in diabetic persons [13,25]. The levels of Pi as well as other metabolic markers potentially related to cancer are also altered in metabolic bone disease [26-28], so that additional adjustment for alkaline phosphatase, a marker of bone turnover, was performed. Our database did not have information regarding phosphate regulators, i.e. vitamin D, FGF23 and parathyroid hormone (PTH) [2,29], but we used season at time of baseline measurement as a proxy for vitamin D [30]. Kidney function is also a potential confounder as renal reabsorption of Pi is a major component in maintaining physiological Pi levels, and kidney disease is a risk factor of cancer [31,32]. Thus, serum creatinine was used in the multivariable models. Further adjustment was done for history of lung disease as a proxy for smoking as the latter has been strongly linked to an increased risk of respiratory tract infection [33] To assess reverse causation [34], we performed a sensitivity analysis in which those with follow-up <3 years were excluded (n = 10,360). Finally, we conducted sex-stratified analyses of Pi and risk of specific cancer sites using quartiles and standardized values of Pi. All analyses were conducted with Statistical Analysis Systems (SAS) release 9.1.3 (SAS Institute, Cary, NC).

## Results

A total of 31,482 persons developed cancer during mean follow-of 12.75 years. Most measurements were taken as part of health examinations done at company health check-ups, so that the majority of the study population (84%) was gainfully employed (Table 1). The age of the participants, serum glucose, alkaline phosphatase and creatinine were higher in the population with cancer than those without cancer. Pi levels were slightly higher in the group without cancer, while no marked difference in calcium levels was noted between the two groups.

**Table 1:** Baseline characteristics of study population

	**N (%)**	
	**No cancer (N=365,810)**	**Cancer (N=31,482)**
**Age (years) -** Mean (SD)	44.00 (14.00)	55.71 (11.93)
**Sex**		
Male	193769 (52.97)	16903 (53.69)
Female	172041 (47.03)	14579 (46.31)
**SES**		
White Collar	132733 (36.28)	12064 (38.32)
Blue Collar	174385 (47.67)	14009 (44.50)
Not gainfully employed or Missing	58692 (16.04)	5409 (17.18)
**Fasting status**		
Fasting	208923 (57.11)	19547 (62.09)
Non-fasting	113613 (31.06)	8153 (25.90)
Missing	43274 (11.83)	3782 (12.01)
**Follow-up time (years) -** Mean (SD)	13.12 (3.89)	8.39 (4.68)
**Pi**^1^**(mmol/L)** Mean (SD)	1.06 (0.17)	1.04 (0.16)
**Calcium (mmol/L)** Mean (SD)	2.39 (0.10)	2.39 (0.10)
**Alkaline phosphatase (mmol/)** Mean (SD)	2.64 (0.96)	2.78 (1.02)
**Glucose (mmol/L) -** Mean (SD)	4.97 (1.29)	5.19 (1.48)
**Creatinine (μmol/L) -** Mean (SD)	81.70 (15.22)	83.77 (16.76)
**Season**		
Winter	93580 (25.58)	8328 (26.45)
Spring	99572 (27.22)	8747 (27.78)
Summer	55799 (15.25)	4601 (14.61)
Fall	116859 (31.95)	9806 (31.15)
**History of diabetes** (ICD-7 260)	1905 (0.52)	201 (0.64)
**History of lung disease** (ICD-7 470–527)	23709 (6.48)	1791 (5.69)

^1^*Pi* inorganic phosphate.

ICD-7, International Classification of Diseases, seventh revision.

Multivariable Cox proportional hazards ratios for quartiles of Pi showed a lower risk of overall cancer for those in the 3^rd^ and 4^th^ quartiles of Pi for both men and women. This pattern of risk was also observed for women, but in men higher quartiles were associated with an increased risk of cancer. When we excluded persons with follow-up <3 years, the positive association between Pi quartiles and overall cancer risk for men weakened slightly (Table 2). When using standardized Pi instead of quartiles, there was a negative association with risk of overall cancer (HR per SD: 0.97 (95% CI: 0.96-0.99), P-value < 0.0001). Excluding the first three years of follow-up did not change the results.

**Table 2:** Hazard ratios and 95% confidence intervals for the risk of overall cancer for quartiles and standardized values of serum Pi levels

	**Overall cancer**	**Overall cancer**^**1**^
	**Hazard ratio (95%CI)**	**Hazard ratio (95%CI)**
**Men and women combined**		
**Standardized Pi** (SD = 0.17)	0.97 (0.96 – 0.99)	0.98 (0.96 – 0.99)
**Quartiles of Pi** (mmol/L)		
< 0.95	1.00 (Ref)	1.00 (Ref)
0.95 – 1.05	0.98 (0.96 – 1.02)	1.00 (0.97 – 1.03)
1.05 – 1.16	0.94 (0.91 – 0.97)	0.95 (0.91 – 0.98)
≥ 1.16	0.94 (0.91 – 0.97)	0.95 (0.92 – 0.98)
P-value for trend	< 0.0001	< 0.0001
**Men**^**2**^		
**Standardized Pi** (SD = 0.17)	1.02 (1.00 – 1.04)	1.02 (1.00 – 1.04)
**Quartiles of Pi** (mmol/L)		
< 0.92	1.00 (Ref)	1.00 (Ref)
0.92 – 1.03	1.06 (1.02 – 1.10)	1.05 (1.01 – 1.09)
1.03 – 1.14	1.04 (1.00 – 1.08)	1.03 (0.98 – 1.08)
≥ 1.14	1.07 (1.03 – 1.12)	1.06 (1.01 – 1.12)
P-value for trend	0.01	0.05
**Women**^**2**^		
**Standardized Pi** (SD = 0.16)	0.97 (0.96 – 0.99)	0.97 (0.95 – 0.99)
**Quartiles of Pi** (mmol/L)		
< 0.99	1.00 (Ref)	1.00 (Ref)
0.99 – 1.09	0.95 (0.91 – 0.99)	0.98 (0.93 – 1.04)
1.09 – 1.19	0.93 (0.89 – 0.97)	0.93 (0.89 – 0.99)
≥ 1.19	0.91 (0.87 – 0.96)	0.93 (0.88 – 0.98)
P-value for trend	< 0.0001	0.001

All models were adjusted for age, sex, SES, fasting status, calcium, alkaline phosphatase, glucose, creatinine, season, history of diabetes and lung diseases.

^1^A sensitivity analysis excluding the first three years of follow-up (n = 386,683).

^2^Not adjusted for sex.

When investigating the relation between quartiles of Pi and risk of different types of cancer in men, we found a statistically significant increase in the risk of pancreatic, lung, thyroid, bone and other cancer in those with higher Pi quartiles (Table 3). Additionally, a higher risk of developing cancer of the liver and gallbladder was found in men in the highest Pi quartile (HR: 1.38 (95% CI: 1.00-1.91) for the fourth quartile of Pi compared to the first). Using standardized Pi, a positive association was also observed between increasing standardized Pi and the risk of non-Hodgkin’s lymphoma in men, but no linear association was observed using quartiles of Pi. However, there was an inverse association between Pi levels and risk of endocrine cancer other than the thyroid gland, prostate, and testis (e.g. HR 0.87 (95% CI: 0.76-1.00) per SD increase of Pi, P-value < 0.0001), although the trend over the quartiles was not linear. There was also a borderline inverse association between standardized Pi and risk of colorectal cancer, but this was not confirmed by Pi quartiles. Excluding other endocrine cancer resulted in a stronger association between Pi and overall cancer in men (e.g. HR: 1.10 (95% CI: 1.03-1.18) per SD increase in Pi, P-value 0.003, results not shown in tables).

**Table 3:** Hazard ratios and 95% confidence intervals for the risk of site-specific cancer for quartiles of serum Pi in men

**Cancer site (ICD-7)**	***n *cases**	**Quartiles of Pi (mmol/L), HR (95% CI)**				**P-value for trend**	**Standardized Pi, HR (95% CI)**
		**< 0.92**	**0.92 – 1.03**	**1.03 – 1.14**	**≥ 1.14**		
**Lip, oral cavity, pharynx (140–149)**	135	1.00 (Ref)	1.12 (0.71 – 1.77)	1.15 (0.71 – 1.86)	1.28 (0.77 – 2.12)	0.31	1.10 (0.93 – 1.30)
**Oesophagus (150)**	196	1.00 (Ref)	1.23 (0.84 – 1.80)	1.26 (0.84 – 1.89)	1.43 (0.94 – 2.18)	0.17	1.12 (0.98 – 1.28)
**Stomach (151)**	400	1.00 (Ref)	1.10 (0.87 – 1.41)	0.80 (0.60 – 1.07)	1.07 (0.80 – 1.44)	0.49	0.98 (0.88 – 1.09)
**Colorectal (153, 154)**	1929	1.00 (Ref)	1.06 (0.94 – 1.18)	0.95 (0.84 – 1.08)	0.91 (0.79 – 1.05)	0.07	0.95 (0.91 – 1.00)
**Liver, gallbladder (155)**	319	1.00 (Ref)	1.15 (0.86 – 1.54)	1.07 (0.78 – 1.47)	1.38 (1.00 – 1.91)	0.14	1.09 (0.98 – 1.22)
**Pancreas (157)**	427	1.00 (Ref)	1.32 (1.03 – 1.71)	1.27 (0.97 – 1.68)	1.41 (1.05 – 1.88)	0.02	1.11 (1.02 – 1.22)
**Larynx (161)**	143	1.00 (Ref)	1.13 (0.72 – 1.77)	1.33 (0.84 – 2.10)	1.28 (0.78 – 2.10)	0.24	1.11 (0.95 – 1.29)
**Lung (162)**	1396	1.00 (Ref)	1.24 (1.07 – 1.42)	1.22 (1.05 – 1.42)	1.35 (1.15 – 1.58)	0.002	1.10 (1.04 – 1.15)
**Prostate (177)**	5075	1.00 (Ref)	1.01 (0.94 – 1.08)	1.02 (0.94 – 1.10)	0.98 (0.90 – 1.07)	0.72	0.98 (0.95 – 1.01)
**Testis (178)**	159	1.00 (Ref)	0.98 (0.60 – 1.58)	0.97 (0.60 – 1.57)	1.11 (0.70 – 1.75)	0.62	1.02 (0.88 – 1.19)
**Kidney (180)**	525	1.00 (Ref)	1.05 (0.84 – 1.32)	1.14 (0.90 – 1.44)	0.87 (0.66 – 1.15)	0.49	1.03 (0.94 – 1.13)
**Bladder (181)**	1181	1.00 (Ref)	0.99 (0.85 – 1.15)	1.04 (0.89 – 1.21)	1.02 (0.86 – 1.21)	0.76	1.02 (0.96 – 1.08)
**Melanoma of skin (190)**	765	1.00 (Ref)	1.02 (0.85 – 1.24)	1.08 (0.89 – 1.32)	1.01 (0.89 – 1.25)	0.66	1.01 (0.94 – 1.09)
**Nonmelanoma of skin (191)**	772	1.00 (Ref)	0.95 (0.80 – 1.14)	0.90 (0.74 – 1.10)	1.13 (0.91 – 1.39)	0.46	0.98 (0.91 – 1.06)
**Brain/central nervous system (193)**	527	1.00 (Ref)	1.03 (0.82 – 1.31)	1.08 (0.84 – 1.37)	1.06 (0.82 – 1.37)	0.74	1.01 (0.92 – 1.10)
**Thyroid gland (194)**	69	1.00 (Ref)	0.94 (0.45 – 1.98)	1.76 (0.90 – 3.43)	1.69 (0.85 – 3.38)	0.04	1.15 (0.93 – 1.41)
**Other endocrine organ (195)**	215	1.00 (Ref)	0.46 (0.31 – 0.66)	0.44 (0.29 – 0.65)	0.67 (0.47 – 0.96)	0.03	0.87 (0.76 – 1.00)
**Bone (196)**	1690	1.00 (Ref)	1.15 (1.02 – 1.31)	1.15 (1.01 – 1.32)	1.25 (1.08 – 1.10)	0.002	1.06 (1.01 – 1.11)
**Soft tissues (197)**	126	1.00 (Ref)	1.48 (0.93 – 2.37)	1.13 (0.67 – 1.92)	1.42 (0.83 – 2.42)	0.32	1.11 (0.95 – 1.30)
**Non-Hodgkin’s lymphoma (200, 202)**	613	1.00 (Ref)	1.24 (1.00 – 1.52)	1.06 (0.84 – 1.33)	1.22 (0.96 – 1.55)	0.25	1.08 (1.00 – 1.17)
**Hodgkin’s lymphoma (201)**	62	1.00 (Ref)	0.98 (0.82 – 1.18)	1.01 (0.99 – 1.02)	1.45 (0.95 – 2.22)	0.51	0.92 (0.71 – 1.20)
**Multiple myeloma (203)**	259	1.00 (Ref)	1.31 (0.96 – 1.77)	0.84 (0.57 – 1.21)	1.20 (0.83 – 1.74)	0.82	1.02 (0.90 – 1.16)
**Leukemia (204–207)**	398	1.00 (Ref)	0.88 (0.68 – 1.13)	0.79 (0.59 – 1.04)	0.85 (0.63 – 1.14)	0.12	0.98 (0.89 – 1.09)
**Other cancer**^**1**^	865	1.00 (Ref)	1.15 (0.96 – 1.38)	1.23 (1.02 – 1.49)	1.38 (1.13 – 1.68)	0.003	1.10 (1.03 – 1.18)

All models were adjusted for age, SES, fasting status, calcium, alkaline phosphatase, glucose, creatinine, season, history of diabetes and lung diseases.

^1^Other cancer than the separately presented sites.

ICD-7, International Classification of Diseases, seventh revision; ref, referent group.

In women, higher Pi quartiles were related with an increased risk of oesophageal, lung, and nonmelanoma skin cancer (Table 4). The test for trend also showed a borderline positive association with risk of laryngeal cancer, but the limited number of cases resulted in low statistical power. Increased risks of stomach and bone cancer were also observed for women in higher quartiles of Pi compared to the first. In contrast, an inverse association was observed between Pi quartiles and risk of breast, endometrial and other endocrine cancers. Furthermore, there was an increased risk of colorectal cancer risk with every SD increase in Pi, although this was not confirmed with Pi quartiles. When cancer of the breast, endometrium, and other endocrine organs were excluded from the analysis, the inverse association between Pi and overall cancer risk in women disappeared (e.g. HR: 1.06 (95% CI: 0.98-1.15) for every SD increase in Pi, P-value 0.13, results not shown in tables).

**Table 4:** Hazard ratios and 95% confidence intervals for the risk of site-specific cancer for quartiles of serum Pi in women

**Cancer site (ICD-7)**	***n *cases**	**Quartiles of Pi (mmol/L), HR (95% CI)**				**P-value for trend**	**Standardized Pi, HR (95% CI)**
		**< 0.99**	**0.99 – 1.09**	**1.09 – 1.19**	**≥ 1.19**		
**Lip, oral cavity, pharynx (140–149)**	66	1.00 (Ref)	0.62 (0.30 – 1.29)	0.93 (0.48 – 1.81)	1.05 (0.55 – 2.00)	0.67	1.04 (0.80 – 1.35)
**Oesophagus (150)**	75	1.00 (Ref)	0.57 (0.25 – 1.30)	1.56 (0.81 – 2.99)	1.95 (1.04 – 3.67)	0.004	1.34 (1.10 – 1.65)
**Stomach (151)**	251	1.00 (Ref)	1.71 (1.18 – 2.47)	1.48 (1.01 – 2.18)	1.52 (1.03 – 2.25)	0.10	1.13 (0.98 – 1.29)
**Colorectal (153, 154)**	1410	1.00 (Ref)	1.06 (0.91 – 1.23)	1.04 (0.89 – 1.21)	1.11 (0.96 – 1.30)	0.22	1.06 (1.00 – 1.12)
**Liver, gallbladder (155)**	260	1.00 (Ref)	1.08 (0.75 – 1.55)	1.38 (0.97 – 1.95)	1.26 (0.88 – 1.81)	0.11	1.09 (0.95 – 1.25)
**Pancreas (157)**	335	1.00 (Ref)	1.08 (0.80 – 1.46)	0.93 (0.67 – 1.28)	1.24 (0.92 – 1.68)	0.28	1.04 (0.93 – 1.18)
**Larynx (161)**	16	1.00 (Ref)	2.86 (0.30 – 27.58)	7.31 (0.90 – 59.67)	5.72 (0.66 – 49.37)	0.05	1.36 (0.85 – 2.17)
**Lung (162)**	887	1.00 (Ref)	1.21 (0.98 – 1.48)	1.51 (1.24 – 1.84)	1.66 (1.37 – 2.02)	< 0.0001	1.20 (1.12 – 1.29)
**Breast (170)**	4925	1.00 (Ref)	0.94 (0.87 – 1.01)	0.89 (0.82 – 0.96)	0.81 (0.75 – 0.88)	< 0.0001	0.93 (0.90 – 0.96)
**Cervix uteri (171)**	318	1.00 (Ref)	1.12 (0.82 – 1.53)	1.09 (0.80 – 1.51)	1.12 (0.82 – 1.54)	0.52	1.03 (0.91 – 1.16)
**Endometrium (172)**	900	1.00 (Ref)	0.83 (0.70 – 0.99)	0.72 (0.60 – 0.87)	0.72 (0.60 – 0.87)	0.0002	0.84 (0.78 – 0.91)
**Ovary (175)**	637	1.00 (Ref)	1.00 (0.81 – 1.24)	0.97 (0.78 – 1.20)	0.90 (0.72 – 1.13)	0.31	0.98 (0.90 – 1.07)
**Other parts of uterus (174, 176)**	67	1.00 (Ref)	0.81 (0.39 – 1.71)	1.37 (0.71 – 2.67)	1.15 (0.58 – 2.29)	0.39	1.07 (0.83 – 1.38)
**Kidney (180)**	262	1.00 (Ref)	1.19 (0.85 – 1.68)	1.24 (0.88 – 1.75)	1.04 (0.72 – 1.49)	0.81	1.02 (0.89 – 1.16)
**Bladder (181)**	325	1.00 (Ref)	1.05 (0.77 – 1.43)	1.11 (0.82 – 1.52)	1.02 (0.74 – 1.40)	0.79	1.02 (0.91 – 1.16)
**Melanoma of skin (190)**	532	1.00 (Ref)	1.02 (0.80 – 1.29)	1.09 (0.86 – 1.39)	0.99 (0.77 – 1.27)	0.93	1.05 (0.95 – 1.15)
**Nonmelanoma of skin (191)**	442	1.00 (Ref)	1.47 (1.12 – 1.94)	1.33 (1.00 – 1.77)	1.53 (1.16 – 2.04)	0.01	1.12 (1.01 – 1.24)
**Brain/central nervous system (193)**	456	1.00 (Ref)	0.90 (0.70 – 1.17)	1.00 (0.78 – 1.30)	0.95 (0.73 – 1.24)	0.88	1.00 (0.90 – 1.11)
**Thyroid gland (194)**	121	1.00 (Ref)	0.77 (0.45 – 1.30)	1.14 (0.70 – 1.85)	0.99 (0.60 – 1.63)	0.65	1.06 (0.88 – 1.27)
**Other endocrine organ (195)**	407	1.00 (Ref)	0.33 (0.25 – 0.43)	0.15 (0.11 – 0.43)	0.19 (0.14 – 0.26)	< 0.0001	0.45 (0.41 – 0.51)
**Bone (196)**	1080	1.00 (Ref)	1.21 (1.02 – 1.44)	1.20 (1.01 – 1.43)	1.19 (1.00 – 1.42)	0.06	1.08 (1.02 – 1.16)
**Soft tissues (197)**	93	1.00 (Ref)	0.84 (0.48 – 1.47)	0.79 (0.44 – 1.41)	1.02 (0.58 – 1.78)	0.96	1.02 (0.82 – 1.29)
**Non-Hodgkin’s lymphoma (200, 202)**	419	1.00 (Ref)	0.96 (0.74 – 1.24)	0.95 (0.72 – 1.24)	0.88 (0.67 – 1.16)	0.38	1.00 (0.90 – 1.11)
**Hodgkin’s lymphoma (201)**	39	1.00 (Ref)	1.23 (0.62 – 2.43)	1.38 (0.70 – 2.75)	0.63 (0.27 – 1.48)	0.94	1.18 (0.86 – 1.62)
**Multiple myeloma (203)**	140	1.00 (Ref)	1.31 (0.82 – 2.10)	1.26 (0.78 – 2.04)	1.14 (0.69 – 1.88)	0.70	1.02 (0.84 – 1.22)
**Leukemia (204–207)**	265	1.00 (Ref)	0.74 (0.51 – 1.06)	0.94 (0.67 – 1.32)	1.26 (0.91 – 1.74)	0.07	1.09 (0.95 – 1.24)
**Other cancer**^**1**^	720	1.00 (Ref)	1.14 (0.92 – 1.40)	1.05 (0.84 – 1.30)	1.20 (0.97 – 1.49)	0.17	1.08 (0.99 – 1.17)

All models were adjusted for age, SES, fasting status, calcium, alkaline phosphatase, glucose, creatinine, season, history of diabetes and lung diseases.

^1^Other cancer than the separately presented sites.

ICD-7, International Classification of Diseases, seventh revision; ref, referent group.

## Discussion

This is the first study evaluating the association between Pi and risk of cancer in a population-based observational setting. We found a positive association between serum Pi and risk of overall cancer in men, but an inverse association for women using data from a large prospective Swedish cohort study. Higher Pi quartiles in men was related to pancreatic, lung, thyroid, bone and other cancers. In women, a positive trend was observed between Pi quartiles and risk of oesophageal, lung, and nonmelanoma skin cancer, while a negative association was seen in breast, endometrial, and other endocrine cancer.

The role of Pi in development of cancer has recently been suggested. Elevated levels of serum Pi were found to enhance gene expression as well as protein translation regulating cell proliferation *in vitro*[3,4]. Furthermore, a high phosphate diet has been reported to promote colonic cell hyperplasia and hyperproliferation in mice, indicating a role of Pi in carcinogenesis [35]. Elevated Pi has been suggested to promote development of cancer via amplifying Akt signaling activities and enhancing cap-dependent translation, eventually resulting in increased cell proliferation [6,36]. On the other hand, also mice treated with low dietary phosphate have been shown to develop increased tumourigenesis and enhanced activities of similar signaling pathways [37]. All these pre-clinical findings suggest that both high and low Pi may influence carcinogenesis.

The present study demonstrated that lower Pi was related to an increased risk of overall cancer in women, while higher Pi levels were linked to increased overall cancer risk in men. These associations remained clear after excluding first three years of follow-up, thus no reverse causation was indicated. Reverse causation between Pi and cancer is plausible since low Pi levels may be caused by increased Pi excretion. The latter is often reported in cancer patients and is suggested to occur through renal proximal tubular dysfunction due to administration of cytotoxic drugs or cancer progression [38]. This was unlikely to be the case in the current study.

In the current study, higher Pi levels were associated with an increased risk of male pancreas, lung, thyroid and bone cancer and female oesophagus, lung, and nomelanoma skin cancer. The consistent positive association between Pi levels and lung cancer corroborated prior biological findings linking high dietary Pi to a significantly increased tumor formation in mouse models of lung cancer [36]. Additionally, elevated serum Pi levels have also been reported to enhance the growth and proliferation of nontumourigenic human bronchial epithelial (NHBE) cells, and this process was linked to increased activation of PI3K/Akt as well as Raf/MEK/ERK pathways which play an important role in carcinogenesis [4]. Nevertheless, when higher Pi doses were administered in similar experiments, a steep decrease in cell growth was observed, indicating the existence of a Pi threshold beyond normal range over which cytotoxicity occurs. Further investigation is necessary to define the acceptable range of Pi levels to maintain physiologic control of cell growth and function.

The observed relation between Pi and nonmelanoma skin cancer in women is also in line with previous experimental findings. In a study by Camalier and colleagues, female mouse models of skin tumorigenesis treated with high dietary Pi showed a 50% increase of tumor formation upon 7, 12-dimethylbenz[a]anthracene/12-O-tetradecanolyphorbol-13-acetate (DMBA/TPA) treatment compared to those treated with low Pi diet [5]. It was suggested that Pi affects the formation of skin tumours partly through increased activation of N-ras and its downstream targets [5]. For cancer of the brain/central nervous system, we observed no clear association with Pi levels, despite the reported effects of Pi on brain growth in animal studies. Jin *et al.* suggested that high dietary Pi reduces brain cell proliferation through suppression of cyclin D1 and PCNA, two marker proteins related to cell cycle [12]. Nevertheless, the same authors also reported increased apoptosis and related disruptions of cell cycle in normal brain cells of mice treated with low dietary Pi [7]. Both low and high levels of Pi are thus likely to impede normal proliferation of brain cells and may also play a role in carcinogenesis. However there is lack of observational studies linking Pi and brain cancer. For colorectal cancer, results in women corroborated the positive link with Pi as shown in experimental findings in mice, but opposing results were found in men [35].

We found a clear inverse association between Pi levels with risks of female breast and endometrial cancers as well as “other endocrine cancers”, which drove the inverse relation with overall cancer risk in women. Breast and endometrial cancers are well-known to be affected by hormonal factors, especially estrogen [39,40]. Increased levels of estrogen are known to negatively regulate circulating Pi, both directly and via modulation of PTH levels [41]. Therefore, it is possible that the inverse association between Pi levels and gynecological cancer risk in women reflects the underlying estrogen levels. Correspondingly, it is suggested that hormonal and metabolic factors regulating Pi, i.e. vitamin D, FGF-23 and PTH, are related to cancer incidence [42-44], and thus their abnormal levels may be responsible for the association between Pi and cancer risks. Finally, the klotho gene encoding the obligate co-receptor for FGF-23 is also a putative tumour suppressor gene [45], further implying the link between Pi regulation and carcinogenesis.

The major strength of this study is the large number of subjects with baseline measurements of serum Pi, all measured in the same laboratory. The use of national registers provided detailed follow-up information on diagnosis of cancer, time of death, and emigration for all subjects. The AMORIS population was mainly selected based on the availability of blood samples from health check-ups in non-hospitalized individuals. However, this healthy cohort effect would not affect the internal validity of the current study and is likely to be minor since it has been shown that the AMORIS cohort is similar to the general working population of Stockholm County in terms of SES and ethnicity [46]. A limitation of this study is that there was no available data on dietary Pi intake or Pi regulators such as FGF23, PTH, and vitamin D [29]. There was no information on other possible confounders such as smoking status and alcohol consumption. History of lung disease was used as a proxy for smoking, however some confounding effect of smoking may remain. For the current study we did not have repeated measurements of phosphate to assess its fluctuations over time. Nonetheless, as alteration in phosphate levels is likely to occur in specific conditions, i.e. kidney disease, ricketts and diabetes, we adjusted the models for these diseases using serum creatinine, alkaline phosphatase, glucose and history of diabetes in order to more accurately reflect phosphate levels. Furthermore, a single measurement of phosphate has been used in many published studies to measure the relation between phosphate metabolism and other diseases [47,48]

## Conclusion

Our findings provide novel epidemiological evidence revealing a decreased cancer risk in women with high Pi and increased risk in men with high Pi. However, women with high Pi displayed a higher risk for developing some specific cancers including oesophageal, lung, and nonmelanoma skin cancer. The persistent negative link between Pi levels and the risk of breast, endometrial and other endocrine cancers which drove the inverse relation between Pi and overall cancer risk in women may imply that Pi rather serves as a proxy for underlying hormonal or metabolic factors instigating carcinogenesis. Further studies in this field should take into account these hormonal and metabolic factors involved in Pi metabolism and also the role of dietary Pi, while also addressing the impacts of other cancer-related effect modifiers beyond the coverage of the current study.

## Competing interest

None declared. Niklas Hammar is employed by the AstraZeneca Sverige, Södertalje, Sweden and the views of the present study are his own and not necessarily any official views of the AstraZeneca Sverige.

## Authors’ contributions

WW designed the study, analyzed the data, interpreted analysis results, and wrote the paper. KM interpreted analysis results and edited the manuscript. HG NH IJ GW LH contributed to the analysis tools and database used in this study and edited the manuscript. MVH conceived and designed the study, interpreted analysis results, and edited the manuscript. All authors read and approved the final manuscript.

## Pre-publication history

The pre-publication history for this paper can be accessed here:

http://www.biomedcentral.com/1471-2407/13/257/prepub
